# 1,7-Bis-(*N*,*N*-dialkylamino)perylene Bisimides: Facile Synthesis and Characterization as Near-Infrared Fluorescent Dyes

**DOI:** 10.3390/ma7117548

**Published:** 2014-11-24

**Authors:** Kew-Yu Chen, Che-Wei Chang

**Affiliations:** Department of Chemical Engineering, Feng Chia University, Taichung 40724, Taiwan; E-Mail: m0111617@fcu.edu.tw

**Keywords:** near-infrared fluorescent dyes, 1,7-bis-(*N*,*N*-dialkylamino)perylene bisimides, intramolecular charge transfer, solvatochromism, Lippert–Mataga equation, density functional theory calculations

## Abstract

Three symmetric alkylamino-substituted perylene bisimides with different *n*-alkyl chain lengths (*n* = 6, 12, or 18), 1,7-bis-(*N*,*N*-dialkylamino)perylene bisimides (**1a**–**1c**), were synthesized under mild condition and were characterized by ^1^H NMR, ^13^C NMR and high resolution mass spectroscopy. Their optical and electrochemical properties were measured using UV-Vis and emission spectroscopic techniques as well as cyclic voltammetry (CV). These compounds show deep green color in both solution and solid state, and are highly soluble in dichloromethane and even in nonpolar solvents such as hexane. The shapes of the absorption spectra of **1a**–**1c** in the solution and solid state were found to be almost the same, indicating that the long alkyl chains could efficiently prevent intermolecular contact and aggregation. They show a unique charge transfer emission in the near-infrared region, of which the peak wavelengths exhibit strong solvatochromism. The dipole moments of the molecules have been estimated using the Lippert–Mataga equation, and upon excitation, they show larger dipole moment changes than that of 1,7-diaminoperylene bisimide (**2**). Moreover, all the dyes exhibit two irreversible one-electron oxidations and two quasi-reversible one-electron reductions in dichloromethane at modest potentials. Complementary density functional theory calculations performed on these chromophores are reported in order to rationalize their electronic structure and optical properties.

## 1. Introduction

Derivatives of perylene bisimide (PBI) have continuously attracted significant attention due to their applications in molecular electronic devices, such as light-emitting diodes [1,2,3,4,5], LCD color filters [6,7], organic field-effect transistors (OFETs) [8,9,10,11,12,13], light-harvesting arrays [14,15], photovoltaic cells [16,17,18,19,20,21,22,23,24,25], molecular wires [26,27], and photochromic materials [28,29]. PBIs have also been utilized as building blocks to construct supramolecular or artificial photosynthetic systems [30,31,32,33]. These organic molecules are advantageous due to their high molar absorptivities, high photochemical and optical stabilities, reversible redox properties, ease of synthetic modification and excellent thermal stability [34,35,36,37,38,39,40,41,42,43,44,45,46,47,48,49,50,51,52,53,54,55]. The electronic characteristics of PBIs can also be fine-tuned by introducing different substituents at the bay-positions (1,6,7,12-positions) of the conjugated perylene core. Based on these rules, a number of perylene bisimide derivatives with either electron-withdrawing or electron-donating groups have been reported in the literature, including: (a) perfluoroalkyl-substituted PBIs [56,57]; (b) cyano-substituted PBIs [58,59], (c) nitro-substituted PBIs [60,61,62]; (d) ferrocenyl-substituted PBIs [63,64], (e) aryl-substituted PBIs [65,66], (f) boryl-substituted PBIs [67]; (g) alkyl-substituted PBIs [68]; (h) piperidinyl-substituted PBIs [69,70,71], (i) pyrrolidinyl-substituted PBIs [72,73,74]; (j) amino-substituted PBIs [75,76], (k) alkylamino-substituted PBIs [77,78,79], (l) alkoxy-substituted PBIs [80,81,82,83,84]; (m) hydroxy-substituted PBIs [85,86], *etc.*

To date, a promising strategy for introducing substituents onto the PBI core is bromination or chlorination of perylene dianhydride. Subsequently, replacement of these halogens is readily executed by traditional substitution reactions or by metal-catalyzed cross-coupling reactions. However, both of these methods are usually accompanied by extensive debromination [77] and stringent reaction conditions such as high temperatures, and absence of oxygen and water. In an effort to expand the scope of PBI-based chromophores available for designing systems for colorful dyes and self-assembly, we synthesized a series of blue dyes based on 1,7-diaminoperylene bisimides [76]. We herein report on the introduction of different long alkyl chains of 1,7-diaminoperylene bisimide (**2**) affording chromophores (**1a**–**1c**) that are deep green in color and that readily undergo two irreversible one-electron oxidations and two quasi-reversible one-electron reductions.

## 2. Experimental Section

### 2.1. General

The starting materials such as perylene-3,4,9,10-tetracarboxyldianhydride, acetic acid, cyclohexylamine, cerium (IV) ammonium nitrate (CAN), tin (II) chloride dihydrate (SnCl_2_.2H_2_O), *N*-methyl-2-pyrrolidinone (NMP), tetrahydrofuran (THF), sodium hydride (NaH), 1-iodohexane (C_6_H_13_I), 1-iodododecane (C_12_H_25_I), and 1-iodooctadecane (C_18_H_37_I) were purchased from Merck (Whitehouse Station, NJ, USA), ACROS (Pittsburgh, PA, USA) and Sigma–Aldrich (St. Louis, MO, USA). Solvents were distilled freshly according to standard procedure. Column chromatography was performed using silica gel Merck Kieselgel *si* 60 (40–63 mesh). ^1^H and ^13^C NMR spectra were recorded in CDCl_3_ on a Bruker 400 MHz NMR spectrometer (Palo Alto, CA, USA). Mass spectra were recorded on a VG70-250S mass spectrometer (Tokyo, Japan). The absorption and emission spectra were measured using a Jasco V-570 UV–Vis spectrophotometer (Tokyo, Japan) and a Hitachi F-7000 fluorescence spectrophotometer (Tokyo, Japan), respectively. Cyclic voltammetry (CV) was performed with a CH instruments (Austin, TX, USA) at a potential rate of 200 mV/s in a 0.1 M solution of tetrabutylammonium hexafluorophosphate (TBAPF_6_) in dichloromethane. Platinum and Ag/AgNO_3_ electrodes were used as counter and reference electrodes, respectively.

### 2.2. Synthesis

#### 2.2.1. Perylene Bisimide (**4**)

A suspension of perylene dianhydride (900 mg, 2.3 mmol), cyclohexylamine (570 mg, 5.8 mmol), and acetic acid (500 mg, 8.3 mmol) in 50 mL of *N*-methyl-2-pyrrolidinone was stirred at 80 °C under nitrogen for 8 h. After the mixture was cooled to room temperature, the precipitate was isolated by filtration, washed with 200 mL of MeOH, and dried in a vacuum. The crude product was purified by silica gel column chromatography with eluent CH_2_Cl_2_ to afford **4** (950 mg, 75%). Characterization data: **4**: ^1^H NMR (400 MHz, CDCl_3_) δ 8.64 (d, *J* = 8.0 Hz, 4H), 8.60 (d, *J* = 8.0 Hz, 4H), 5.05 (m, 2H), 2.58 (m, 4H), 1.91 (m, 4H), 1.76 (m, 6H), 1.36–1.46 (m, 6H). MS (FAB): m/z (relative intensity) 555 (M^+^, 100); HRMS calcd. for C_36_H_31_N_2_O_4_ 555.2284, found 555.2290.

#### 2.2.2. Synthesis of 1,7-Dinitroperylene Bisimide (**3**)

A mixture of perylene bisimide **4** (900 mg, 1.6 mmol), cerium (IV) ammonium nitrate (CAN) (2.4 g, 4.4 mmol), nitric acid (0.1 M, 6.0 mL) and dichloromethane (150 mL) was stirred at 25 °C under N_2_ for 48 h. The mixture was neutralized with 10% KOH and extracted with CH_2_Cl_2_. After solvent was removed, the crude product was purified by silica gel column chromatography with eluent CH_2_Cl_2_ to afford **3** (837 mg, 80%). Characterization data: **3**: ^1^H NMR (400 MHz, CDCl_3_) δ 8.78 (s, 2H), 8.67 (d, *J =* 8.0 Hz, 2H), 8.28 (d, *J =* 8.0 Hz, 2H), 4.99 (m, 2H), 2.51 (m, 4H), 1.92 (m, 4H), 1.74 (m, 6H), 1.46 (m, 4H), 1.36 (m, 2H); MS (FAB): m/z (relative intensity) 645 (M+H^+^, 100); HRMS calcd. for C_36_H_29_O_8_N_4_ 645.1985, found 645.1981.

#### 2.2.3. Synthesis of 1,7-Diaminoperylene Bisimide (**2**)

Tin chloride dihydrate (1.5 g, 7.2 mmol) and **3** (0.8 g, 1.2 mmol) were suspended in 60 mL of THF, and stirred 20 min. The solvent was refluxed with stirring for 6 h at 80 °C. THF was removed at the rotary evaporator, and the residue was dissolved in ethyl acetate and washed with 10% NaOH solution and brine. The organic layer was dried over anhydrous MgSO_4_ and the filtrate was concentrated under reduced pressure. The crude product was purified by silica gel column chromatography with eluent ethyl acetate/n-hexane (2/3) to afford **2** (595 mg, 85%). Characterization data: **2**: ^1^H NMR (400 MHz, CDCl_3_) δ 8.87 (d, *J* = 8.4 Hz, 2H), 8.43 (d, *J* = 8.4 Hz, 2H), 8.14 (s, 2H), 5.04, (m, 2H), 4.94 (s, 4H), 2.61 (m, 4H), 1.93 (m, 4H), 1.74 (m, 6H), 1.36–1.54 (m, 6H); MS (FAB): m/z (relative intensity) 585 (M+H^+^, 100); HRMS calcd. for C_36_H_3__3_O_4_N_4_ 585.2502, found 585.2504.

#### 2.2.4. General Procedure for Alkylation (**1a**–**1c**)

A mixture of solution of **2** (410 mg, 0.70 mmol), sodium hydride (97%, 200 mg, 8.00 mmol) and dry THF (60 mL) was stirred at 0 °C under N_2_ for 30 min. Alkyl iodide (4.20 mmol) was then added and the resulting mixture was stirred for 8 h. The resulting mixture was diluted with 15 mL of water and extracted with CH_2_Cl_2_. The crude product was purified by silica gel column chromatography with eluent ethyl acetate/n-hexane (1/2) to afford **1a** (**1b** or **1c**) in 75% yield. Characterization data: **1a**: ^1^H NMR (400 MHz, CDCl_3_) δ 9.21 (d, *J* = 8.0 Hz, 2H), 8.45 (s, 2H), 8.38 (d, *J* = 8.0 Hz, 2H), 5.03 (m, 2H), 3.45 (m, 4H), 3.15 (m, 4H), 2.57 (m, 4H), 1.87 (m, 4H), 1.15–1.75 (m, 44H), 0.82 (t, *J* = 6.4 Hz, 12H); ^13^C NMR (100 MHz, CDCl_3_) δ 164.51, 164.21, 148.51, 135.42, 130.32, 128.21, 125.31, 124.29, 122.90, 122.74, 122.63, 121.10, 53.77, 52.54, 31.42, 29.17, 27.46, 26.89, 26.60, 25.52, 22.49, 13.90; MS (FAB): m/z (relative intensity) 921 (M+H^+^, 100); HRMS calcd. for C_60_H_81_O_4_N_4_ 921.6256, found 921.6250. Selected data for **1b**: ^1^H NMR (400 MHz, CDCl_3_)δ 9.20 (d, *J* = 8.4 Hz, 2H), 8.45 (s, 2H), 8.37 (d, *J* = 8.4 Hz, 2H), 5.04 (m, 2H), 3.46 (m, 4H), 3.17 (m, 4H), 2.60 (m, 4H), 1.87 (m, 4H), 1.12–1.75 (m, 92H), 0.85 (t, *J* = 6.5 Hz, 12H); ^13^C NMR (100 MHz, CDCl_3_) δ 164.40, 164.06, 148.46, 135.32, 130.25, 128.13, 125.25, 124.23, 122.81, 122.70, 122.59, 121.05, 53.71, 52.43, 31.81, 29.53, 29.45, 29.24, 29.21, 29.12, 27.46, 27.16, 26.55, 25.47, 22.59, 14.01; MS (FAB): m/z (relative intensity) 1258 (M+H^+^, 100); HRMS calcd. for C_84_H_129_O_4_N_4_ 1258.0014, found 1258.0004. Selected data for **1c**: ^1^H NMR (400 MHz, CDCl_3_)δ 9.19 (d, *J* = 8.4 Hz, 2H), 8.45 (s, 2H), 8.38 (d, *J* = 8.4 Hz, 2H), 5.04 (m, 2H), 3.45 (m, 4H), 3.15 (m, 4H), 2.60 (m, 4H), 1.87 (m, 4H), 1.14–1.74 (m, 140H), 0.86 (t, *J* = 6.4 Hz, 12H); ^13^C NMR (100 MHz, CDCl_3_) δ 164.45, 164.12, 148.51, 135.39, 130.32, 128.20, 125.30, 124.30, 122.89, 122.76, 122.66, 121.12, 53.77, 52.51, 31.91, 29.68, 29.52, 29.34, 29.27, 29.19, 27.53, 27.23, 26.60, 25.53, 22.66, 14.07; MS (FAB): m/z (relative intensity) 1595 (M+H^+^, 100); HRMS calcd. for C_108_H_177_O_4_N_4_ 1595.3803, found 1595.3815.

## 3. Results and Discussion

### 3.1. Synthesis

**Scheme 1:**
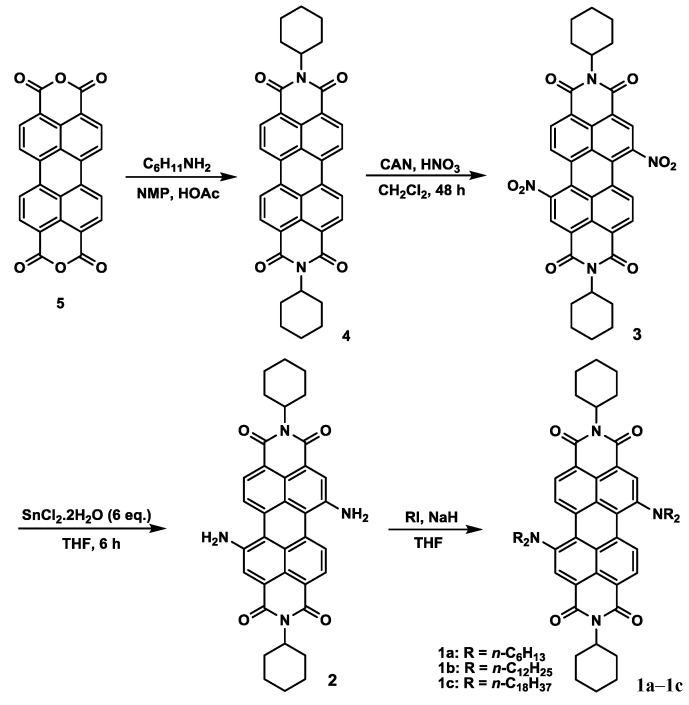
The synthetic route for **1a**–**1c**.

**Figure 1:**
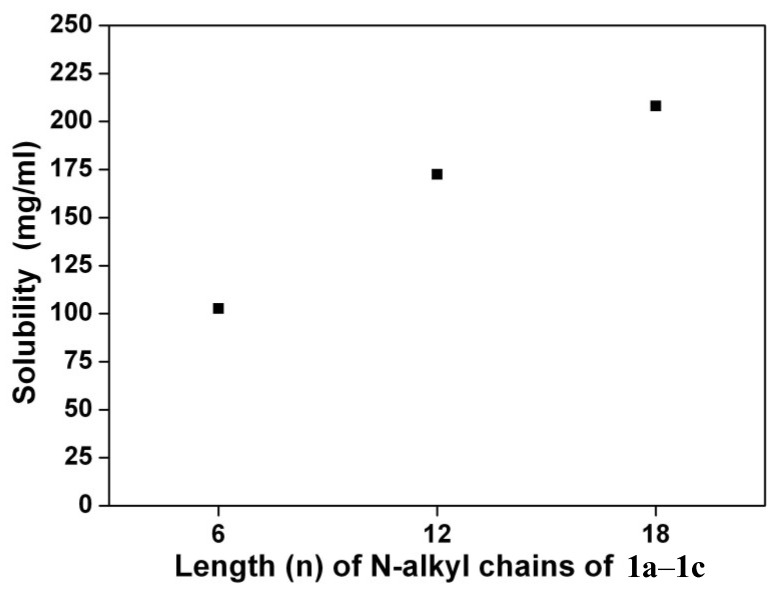
Solubility of **1a**–**1c** in dichloromethane (25 °C).

**Figure 2:**
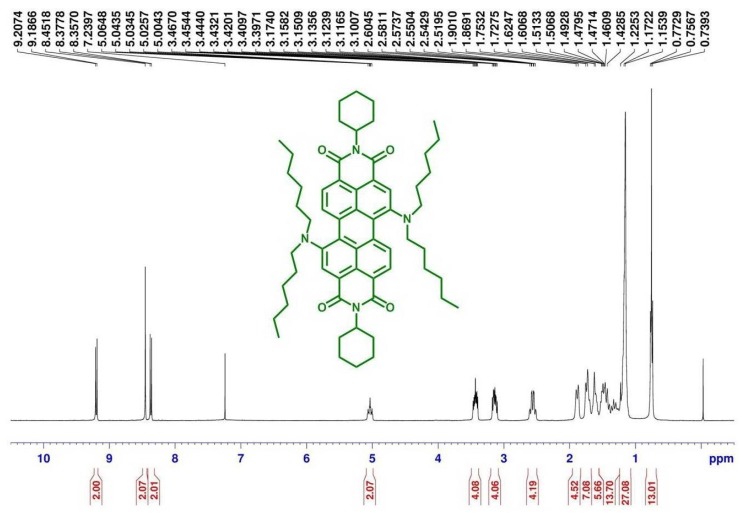
^1^H NMR (400 MHz, CDCl_3_) spectra of **1a**.

Scheme 1 depicts the chemical structures and synthetic routes of symmetric 1,7-dialkylamino substituted PBIs (**1a**–**1c**). Synthesis starts from an imidization [87] of perylene dianhydride (**5**) by reaction with cyclohexylamine (C_6_H_11_NH_2_). The dinitration can then be achieved by a reaction of perylene bisimide (**4**) with cerium (IV) ammonium nitrate (CAN) and HNO_3_ under ambient temperature for 48 h [60], giving 1,6- and 1,7-dinitroperylene bisimides in high yields of *ca.* 80%. The regioisomeric 1,6- and 1,7-dinitroperylene bisimides can be successfully separated by high performance liquid chromatography (HPLC). Pure 1,7-regioisomer (**3**) can also be obtained through repetitive crystallizations. The reduction of 1,7-dinitroperylene bisimide (**3**) by tin (II) chloride dihydrate (SnCl_2_.2H_2_O) in refluxing THF obtained 1,7-diaminoperylene bisimide (**2**). Finally, three 1,7-dialkylamino substituted perylene bisimide derivatives (**1a**–**1c**) with different *n*-alkyl chain lengths (*n* = 6, 12, or 18) can be synthesized by the alkylation of **2** with the corresponding alkyl halides. These compounds show deep green color in both solution and solid state, and are highly soluble in dichloromethane (Figure 1) and even in nonpolar solvents such as hexane. The symmetric structure of 1,7-bis-(*N*,*N*-dialkylamino)perylene bisimides (**1a**–**1c**) can be verified by the presence of three signals (one singlet and two doublet signals) at δ 8.3–9.3 ppm in the ^1^H NMR spectrum, which indicates that there are only three different kinds of protons in the conjugated perylene core (Figure 2). Detailed synthetic procedures and product characterization are provided in the Experimental Section and Supplementary Materials (Figures S1–S6).

### 3.2. Optical Properties

**Figure 3:**
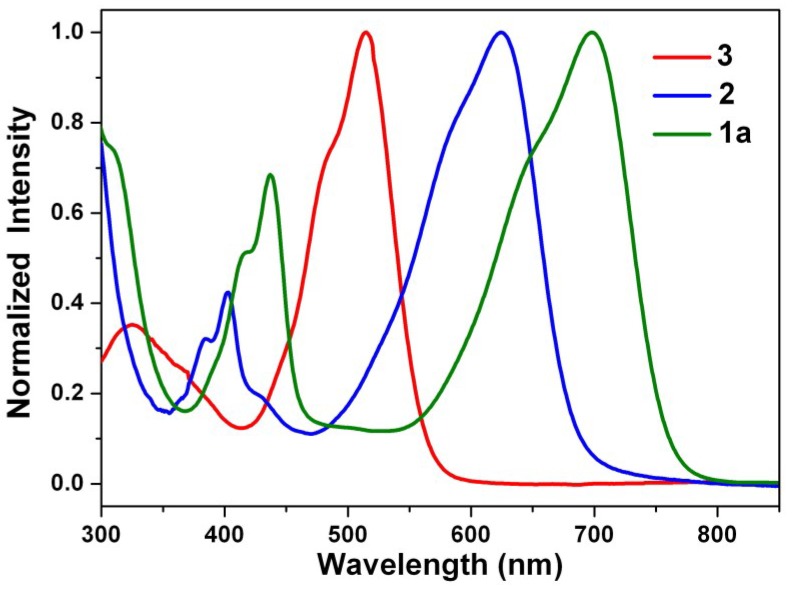
Normalized absorption spectra of **1a**, **2**, and **3** in dichloromethane solution.

Figure 3 shows the steady state absorption spectra of the green dye **1a**, the blue dye **2**, and the red dye **3** in dichloromethane. The spectra of **1b** and **1c** can be found in the Supplementary Materials (Figures S7 and S8). The absorption spectrum of 1,7-dinitroperylene bisimide (**3**) is nearly identical with the spectrum of the non-substituted perylene bisimide (**4**), but they do not show fluorescence [60]. On the other hand, the reduction of **3** to **2** switches the substituents from electron-withdrawing nitro groups to electron-donating amino groups and causes a distinct red shift. The spectra of 1,7-diamino substituted (**2**) and 1,7-dialkylamino substituted (**1a**–**1c**) PBIs are dominated by very broad absorption bands that cover a large part of the visible spectrum (350–800 nm). These broad bands are typical for perylene bisimide derivatives *N*-substituted at the bay-core positions, due to charge transfer absorption [77]. The longest wavelength absorption band of 1,7-diaminoperylene bisimide (**2**: 620 nm) is red-shifted relative to that of 1,7-dinitroperylene bisimide (**3**: 515 nm), but it is blue-shifted relative to that of 1,7-dialkylaminoperylene bisimide (**1a**: 698 nm). It appears that the inductive effect of the alkyl groups in **1a**–**1c** causes an additional red shift. Additionally, the longest wavelength absorption band of **1a**–**1c** exhibits a red shift when the solvent polarity increases (Table 1), which is consistent with previous studies [75].

**Table 1:** Summary of optical absorption and emission properties of **1a**–**1c** in various solvents.

1a/1b/1c	λ_abs_ (nm) ^a^	λ_em_ (nm) ^a^	Stokes shift (nm)	Φ ^b^ × 10^2^
cyclohexane	667/670/670	711/714/716	44/44/48	3.03/4.74/3.14
diethyl ether	675/676/676	726/725/726	51/46/51	0.44/0.80/0.92
ethyl acetate	687/688/687	741/740/740	57/54/55	0.22/0.41/0.42
dichloromethane	698/702/701	755/758/758	52/55/54	0.20/0.40/0.41
acetonitrile	699/703/703	760/760/761	61/56/57	0.25/0.26/0.26

^a^ Measured at 2 × 10^−5^ M; ^b^ Determined with *N*,*N*’-dioctyl-3,4,9,10-perylenedicarboximide as reference [31].

**Figure 4:**
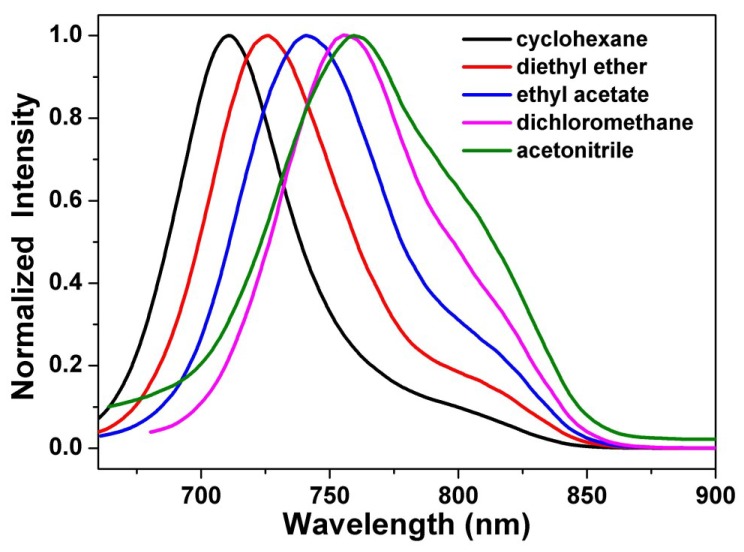
Normalized emission spectra of **1a** in various solvents.

Figure 4 depicts the steady state emission spectra of **1a** in solvents of varying polarity, where those of **1b** and **1c** can be found in the Supplementary Materials (Figures S9 and S10). Unlike the small shift in absorption spectra, the fluorescence spectra of **1a**–**1c** are largely red-shifted if there is any increase of the solvent polarity, which indicates strong intramolecular charge transfer characteristics for the excited states of **1a**–**1c** (Table 1). Using the well-established fluorescence solvatochromic shift method [88], we measured the stabilization of the excited-states of **1a**–**1c** and compared these results to those of **2**. The change of magnitudes for dipole moments between ground and excited states, *i.e.*, $\Delta \mu =\left|{\vec{\mu }}_{e}-{\vec{\mu }}_{g}\right|$, can be calculated by the Lippert–Mataga equation and expressed as:

(1) $${\bar{v}}_{a}-{\bar{v}}_{f}=\frac{\text{2}}{hc}{\left({\vec{\mu }}_{e}-{\vec{\mu }}_{g}\right)}^{2}a_{0}^{-3}+\text{const.}$$

where *h* is the Planck constant, *c* is the speed of light, and $a_{0}$ denotes the cavity radius in which the solute resides, ${\bar{v}}_{a}-{\bar{v}}_{f}$ is the Stokes shift of the absorption and emission peak maximum, and Δf is the orientation polarizability defined as:

(2) $$\Delta f=f\left(\varepsilon \right)-f\left(n^{2}\right)=\frac{\varepsilon -1}{2\varepsilon +1}-\frac{n^{2}-1}{2n^{2}+1}$$

**Figure 5:**
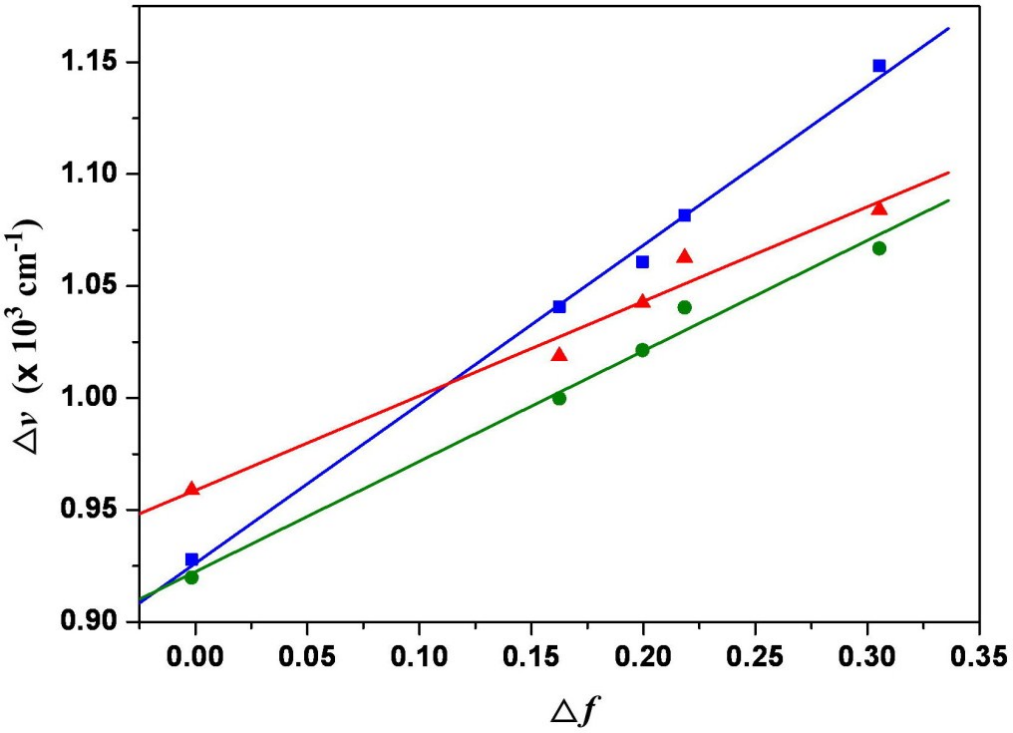
Lippert–Mataga plots for **1a** (blue line), **1b** (green line), and **1c** (red line). The solvents from left to right are (1) cyclohexane; (2) diethyl ether; (3) ethyl acetate; (4) dichloromethane, (5) acetonitrile.

The plot of the Stokes shift ${\bar{\upsilon }}_{a}-{\bar{\upsilon }}_{f}$ as a function of Δf is sufficiently linear for **1a**–**1c** (Figure 5). Accordingly, $\Delta \mu =\left|{\vec{\mu }}_{e}-{\vec{\mu }}_{g}\right|$ values can be estimated as 7.9 D, 9.1 D and 9.7 D for **1a**–**1c**. These values indicate that the 1,7-dialkyamino-substituted PBIs (**1a**–**1c**) have larger dipole moment changes than that (7.4 D) of the 1,7-diamino-substituted compound (**2**).

### 3.3. Quantum Chemistry Computation

**Figure 6:**
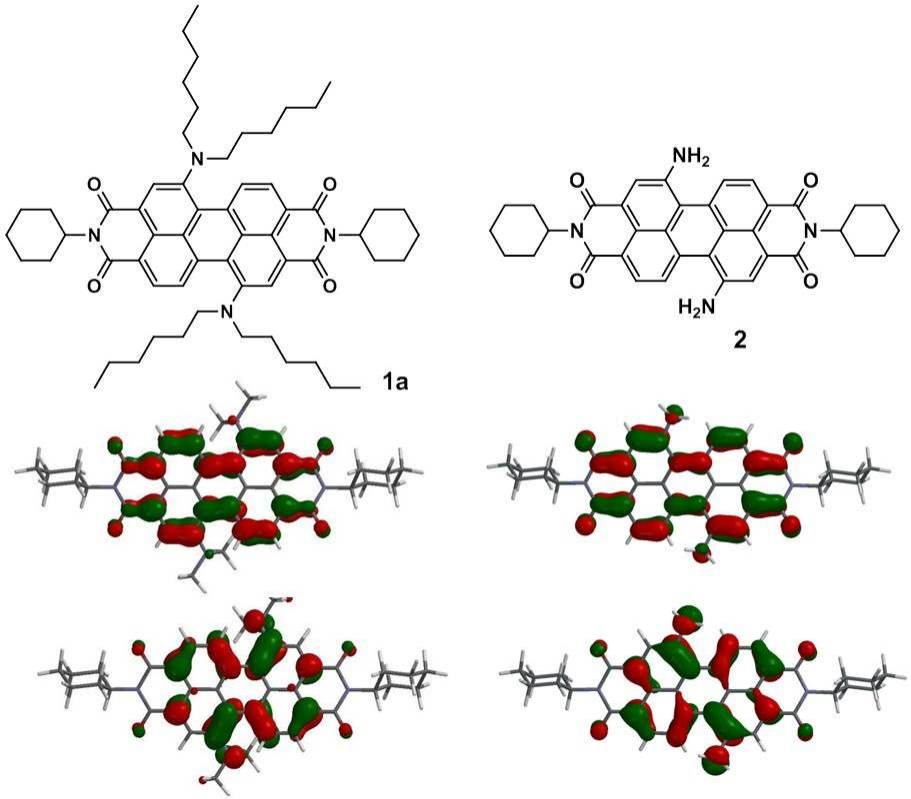
Calculated frontier orbitals for **1a** and **2**. The upper structures show the lowest unoccupied molecular (LUMOs) and the lower ones show the highest occupied molecular orbitals (HOMOs). Methyl groups replace the hexyl groups for clarity.

To gain better insight into the molecular structures and electronic properties of **1a**–**1c**, quantum chemical calculations were performed using density functional theory (DFT) at the B3LYP/6-31G** level. Figure 6 shows the highest occupied molecular orbitals (HOMOs) and the lowest unoccupied molecular orbitals (LUMOs) of **1a** and **2**. The HOMO of all amino-substituted PBIs (**1a**–**1c** and **2**) is delocalized chiefly on the amino group and the perylene core, while the LUMO is extended from the central perylene core to the bisimide groups. Table 2 summarizes the calculated and experimental parameters for perylene bisimide derivatives **1a**–**1c**. Obviously, the HOMO/LUMO energy levels of **1a**–**1c** and **2** are higher than those of **3** and **4**, which can be explained by the fact that the amino substituent is a strong electron-donating group and hence increases both the HOMO and LUMO energy levels. Furthermore, the calculated HOMO–LUMO band gap energies of **1a**–**1c** are in good agreement with the experimental data (Table 2).

**Figure 7:**
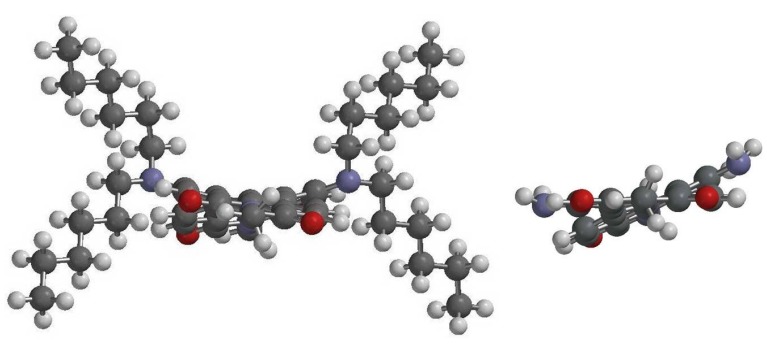
DFT (B3LYP/6-31G**) geometry-optimized structures of **1a** (left) and **2** (right) shown with view along the long axis. For computational purposes, methyl groups replace the cyclohexyl groups at the imide positions.

DFT calculations also demonstrate that the ground-state geometries of the perylene core have different core twist angles (Figure 7 and Table 2), *i.e.*, approximate dihedral angles between the two naphthalene subunits attached to the central benzene ring; these are ~17.53° and ~17.54° for **1a**, ~17.55° and ~17.57° for **1b**,~17.58° and ~17.59° for **1c**, ~19.21° and ~19.43° for **2**, and ~17.02° and ~17.12° for **3**, and all are larger than those of **4** (~0.00°). As a whole, the core twist angles of the diamino-substituted PBIs (**1** and **2**) are slightly larger than that of the dinitro-substituted compound (**3**).

**Table 2:** Calculated and experimental parameters for perylene bisimide derivatives.

Compound	HOMO ^a^	LUMO ^a^	*E*_g_^a^	*E*_g_^b^	*μ*_g_^c^	*μ*_e_^d^	Twisting angle (°)
**1a**	−5.24	−3.12	2.12	1.76	2.9	10.8	17.53, 17.54
**1b**	−5.24	−3.12	2.12	1.76	3.0	12.1	17.55, 17.57
**1c**	−5.23	−3.11	2.12	1.77	3.0	12.7	17.58, 17.59
**2**	−5.33	−3.05	2.28	2.14	2.6	7.8	19.21, 19.43
**3**	−6.57	−4.11	2.46	2.40	–	–	17.02, 17.12
**4**	−5.94	−3.46	2.48	2.38	–	–	0.00, 0.00

^a^ Calculated by DFT/B3LYP (in eV); ^b^ At absorption maxima (*E*_g_ = 1240/λ_max_, in eV); ^c^ Ground-state dipole moment (calculated by DFT/B3LYP, in Debye); ^d^ Excited-state dipole moment (in Debye).

### 3.4. Electrochemical Properties

**Figure 8:**
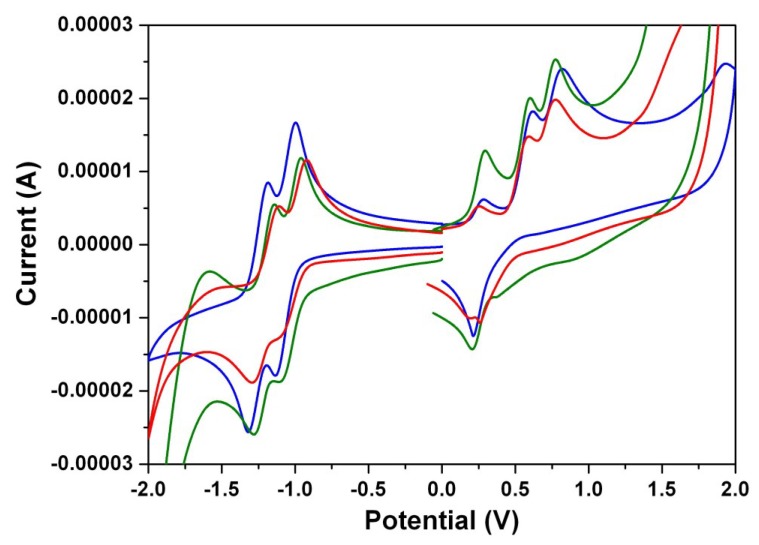
The cyclic voltammograms of **1a** (blue line), **1b** (green line), and **1c** (red line) measured in dichloromethane solution with ferrocenium/ferrocene as an internal standard, at 200 mV/s.

Figure 8 shows the cyclic voltammograms of **1a**–**1c**. These dyes undergo two irreversible one-electron oxidations and two quasi-reversible one-electron reductions in dichloromethane at modest potentials. Table 3 summarizes the redox potentials and the HOMO and LUMO energy levels estimated from cyclic voltammetry (CV) for **1a**–**1c**. It is apparent that both the first oxidation and the first reduction potentials are shifted toward more negative (positive) values with introducing strongly electron-donating (electron-withdrawing) groups onto the perylene core, while both the HOMO and LUMO energy levels increase (decrease) with the trend. The HOMO/LUMO energy levels of **1a**, **1b**, **1c**, and **2** are estimated to be −5.25/−3.49, −5.23/−3.47, −5.22/−3.45, and −5.39/−3.25 eV, respectively. The HOMO–LUMO energy gaps of **1a**–**1c** are found to be almost the same, which indicates that different *N*-alkyl chain lengths do not significantly affect the band gap energies.

**Table 3:** Summary of half-wave redox potentials, HOMO and LUMO energy levels for perylene bisimide derivatives.

Compound	*E*^+^_1/2_^a^	*E*^2+^_1/2_^a^	*E*^−^_1/2_^a^	*E*^2−^_1/2_^a^	HOMO ^b^	LUMO ^b^
**1a**	0.62	0.82	−1.06	−1.22	−5.25	−3.49
**1b**	0.60	0.77	−1.04	−1.20	−5.23	−3.47
**1c**	0.59	0.77	−1.02	−1.19	−5.22	−3.45
**2**	0.79	1.17	−1.15	−1.24	−5.39	−3.25
**3 ^c^**	−	−	−0.09	−0.34	−6.75	−4.35
**4 ^c^**	−	−	−0.46	−0.76	−6.36	−3.98

^a^ Measured in a solution of 0.1 M tetrabutylammonium hexafluorophosphate (TBAPF_6_) in dichloromethane *versus* SCE (in V); ^b^ Calculated from *E*_HOMO_ = −4.88 – (*E*_oxd_ −*E*_Fc/Fc+_), *E*_LUMO_ = *E*_HOMO_ + *E*_g_; ^c^ Estimated *versus* vacuum level from *E*_LUMO_ = −4.44 – *E*_(1)_.

### 3.5. Stacking Behaviors of Dyes in Solution and Solid State

**Figure 9:**
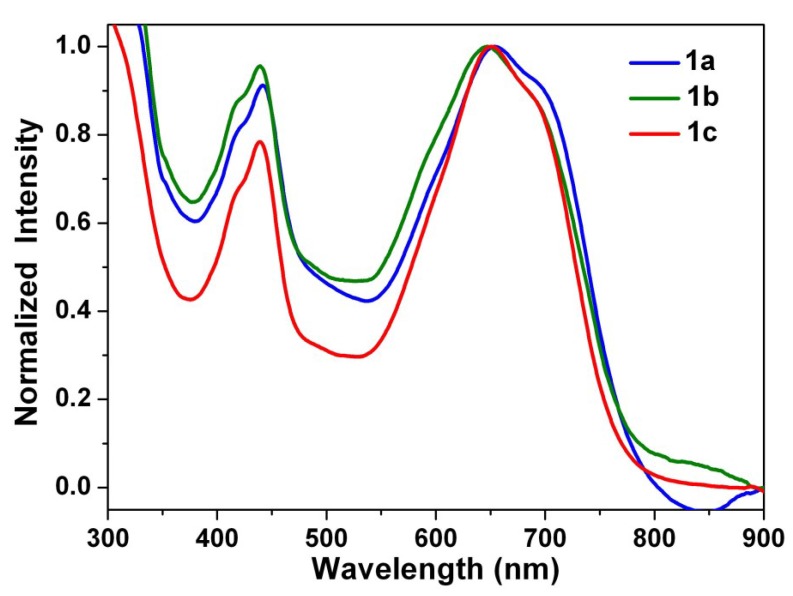
Normalized absorption spectra of **1a**–**1c** in neat film.

Figure 9 depicts the absorption spectra recorded for thin drop-cast films of **1a**–**1c**. The shapes of the absorption spectra of **1a**–**1c** in solid state and in solution are found to be almost the same in view of wavelength range (absorption of up to 800 nm for **1a**–**1c**) and peak positions, which indicates that it is difficult for **1a**–**1c** to form π-aggregates. Thus, we can ascertain that the long alkyl chains not only largely increases the solubility of **1a**–**1c** compared with **2**, but also efficiently reduces intermolecular contact and aggregation.

## 4. Conclusions

We have successfully synthesized three green dyes based on 1,7-dialkylamino substituted PBIs (**1a**–**1c**). All the new PBI dyes are highly soluble in dichloromethane and even in nonpolar solvents such as hexane. The shapes of the absorption spectra of **1a**–**1c** in solution and solid state are found to be virtually the same, which indicates that the long alkyl chains can efficiently prevent intermolecular contact and aggregation. They exhibit a unique charge transfer emission in the near-infrared region, of which the peak wavelengths exhibit strong solvatochromism. Upon excitation, they show larger dipole moment changes than that of **2**; the dipole moments of these compounds have been estimated using DFT calculations and the Lippert–Mataga equation. In addition, they undergo two irreversible one-electron oxidations and two quasi-reversible one-electron reductions in dichloromethane at modest potentials. Research on their applications to near-infrared fluorescence imaging [89,90,91] is currently in progress.

## Supplementary Materials

Supplementary materials can be accessed at: http://www.mdpi.com/1996-1944/7/11/7548/s1.
