# Supplementary material for: 1,7-Bis-(N,N-dialkylamino)perylene Bisimides: Facile Synthesis and Characterization as Near-Infrared Fluorescent Dyes
Source: Materials (Basel). 2014 Nov 24;7(11):7548–65. doi: 10.3390/ma7117548 (PMC5512673; doi:10.3390/ma7117548)

# Supplementary Materials

**Figure S1.** Normalized absorption spectra of **1b** in various solvents.

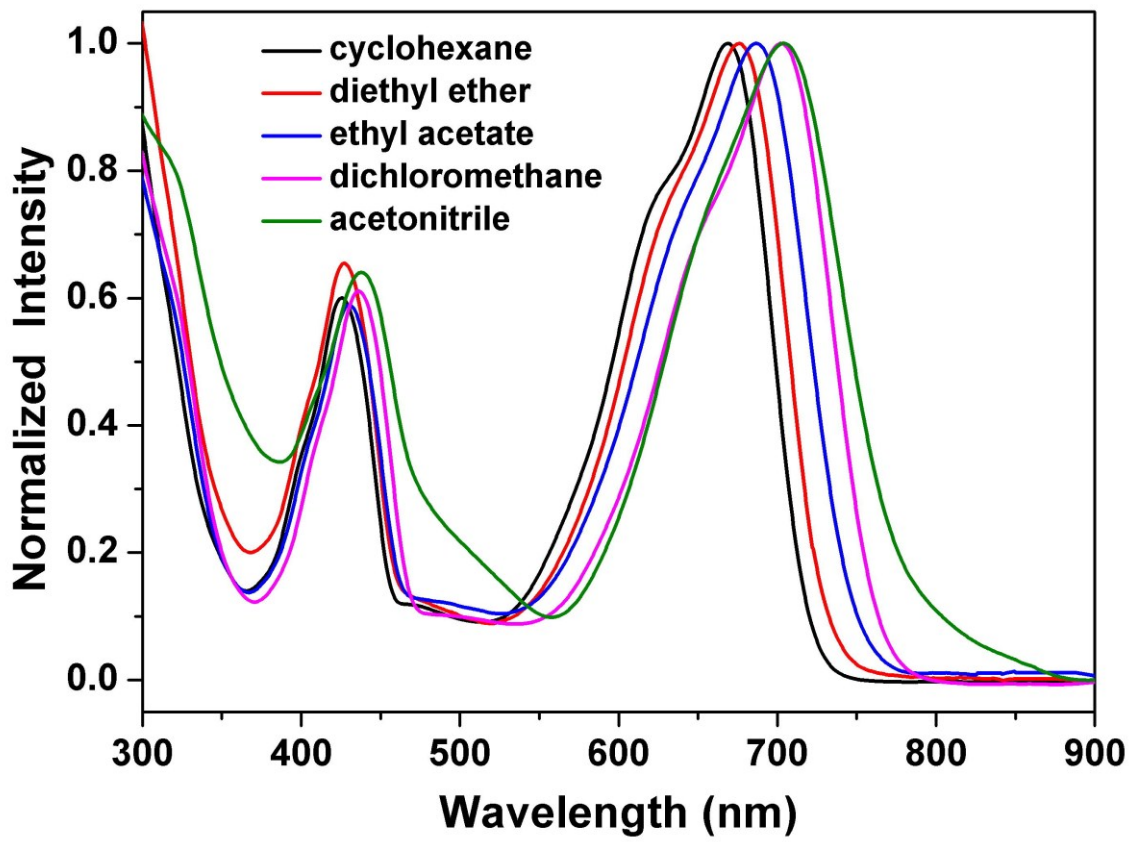


**Figure S2.** Normalized emission spectra of **1b** in various solvents.

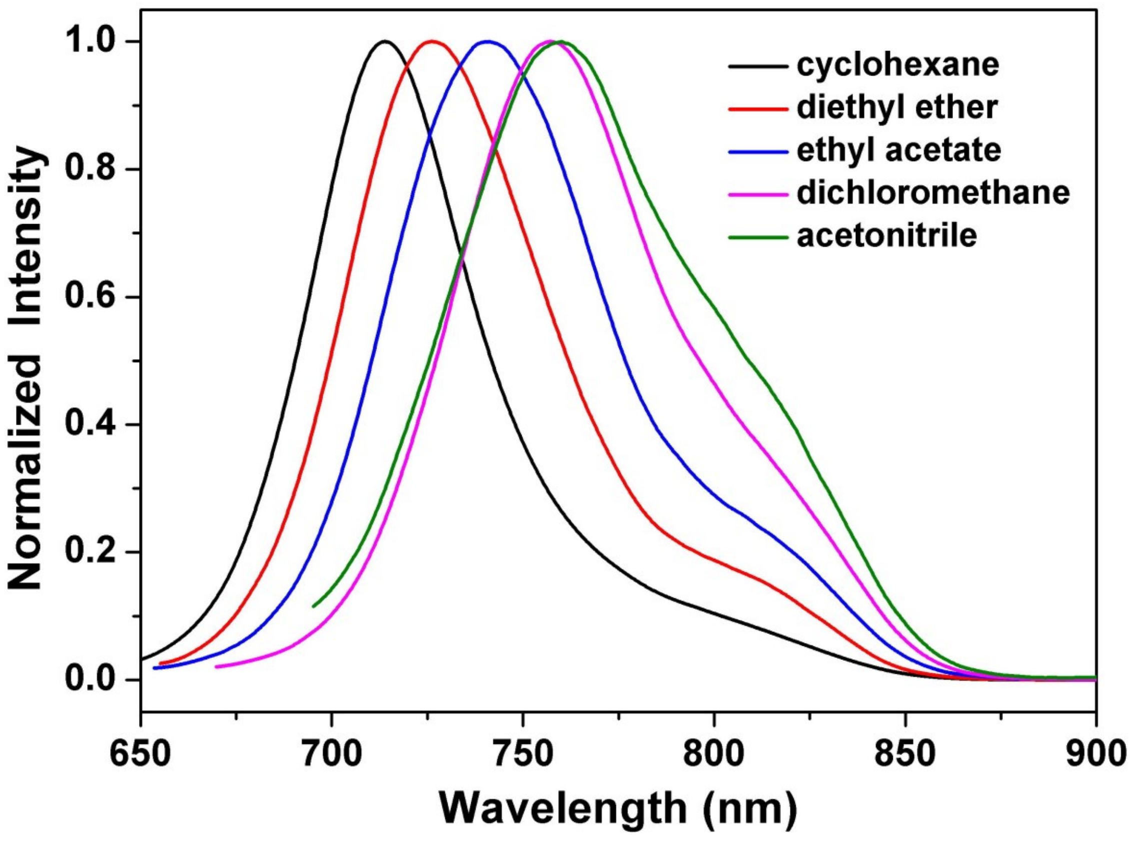

**Figure S3.** Normalized absorption spectra of **1c** in various solvents.

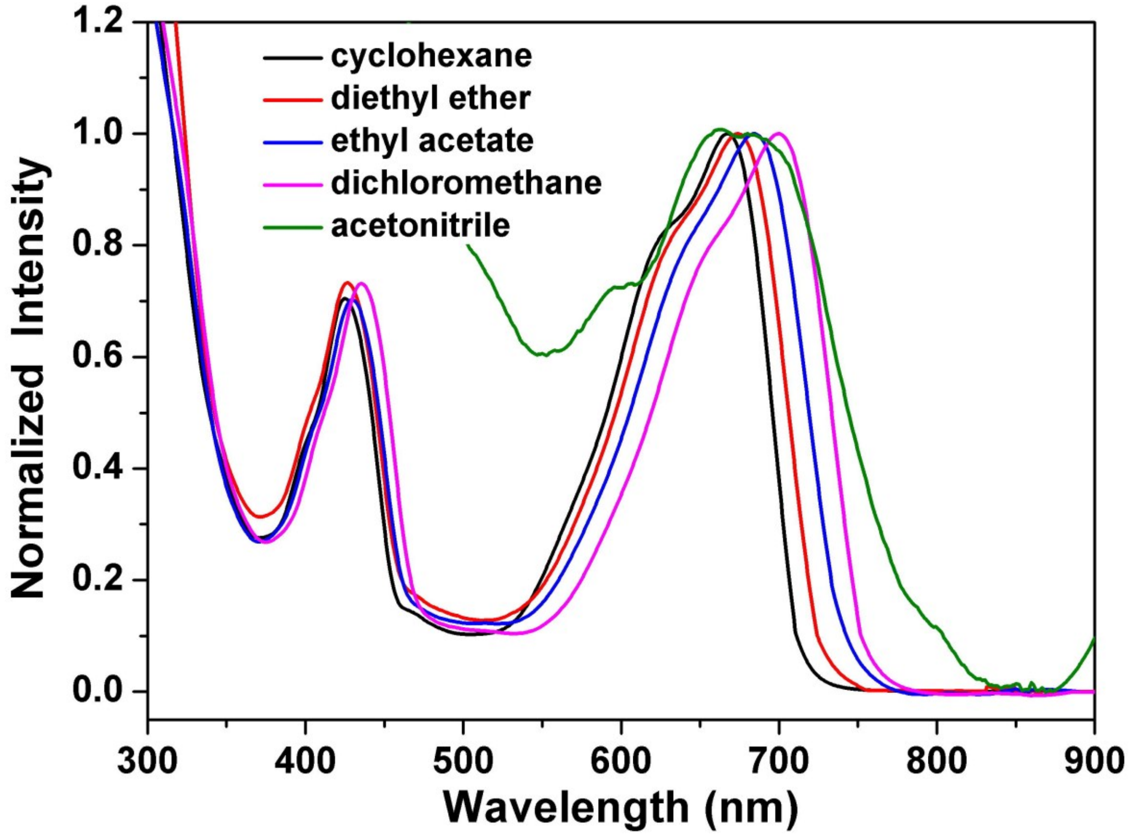


**Figure S4.** Normalized emission spectra of **1c** in various solvents.

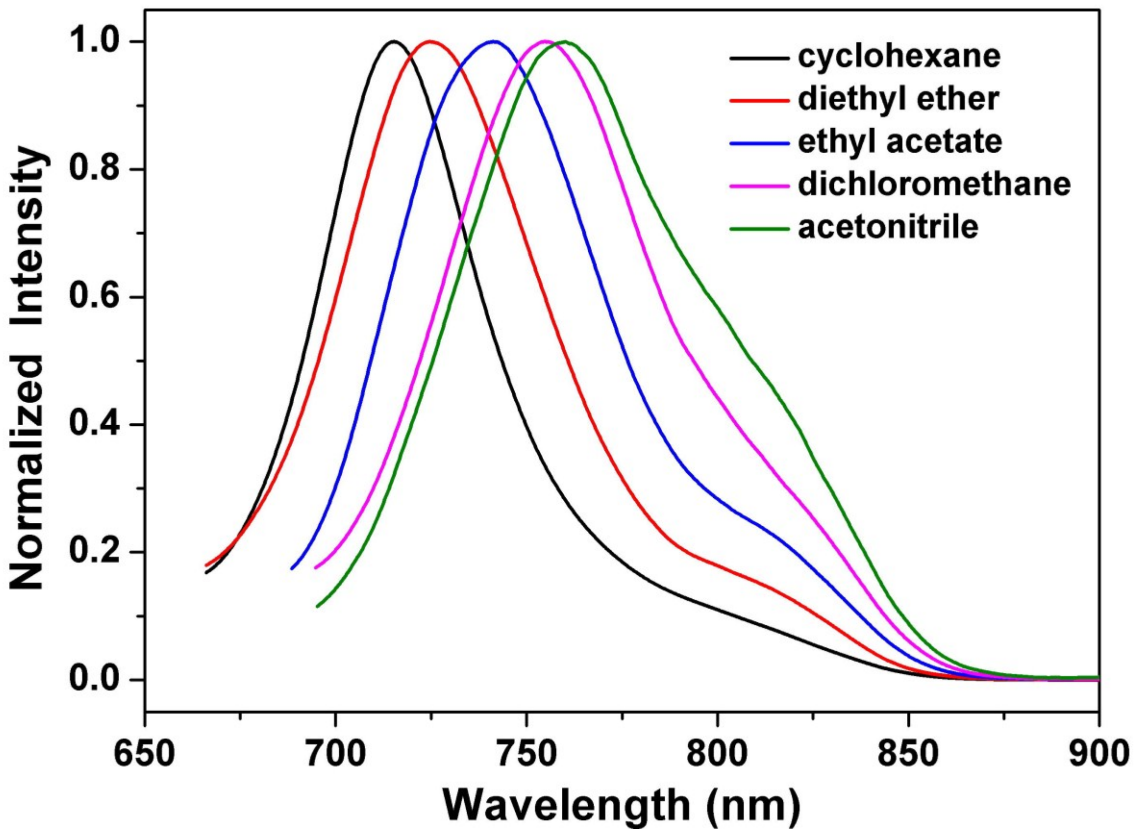

**Figure S5.** $^{1}$H NMR of **1a**.

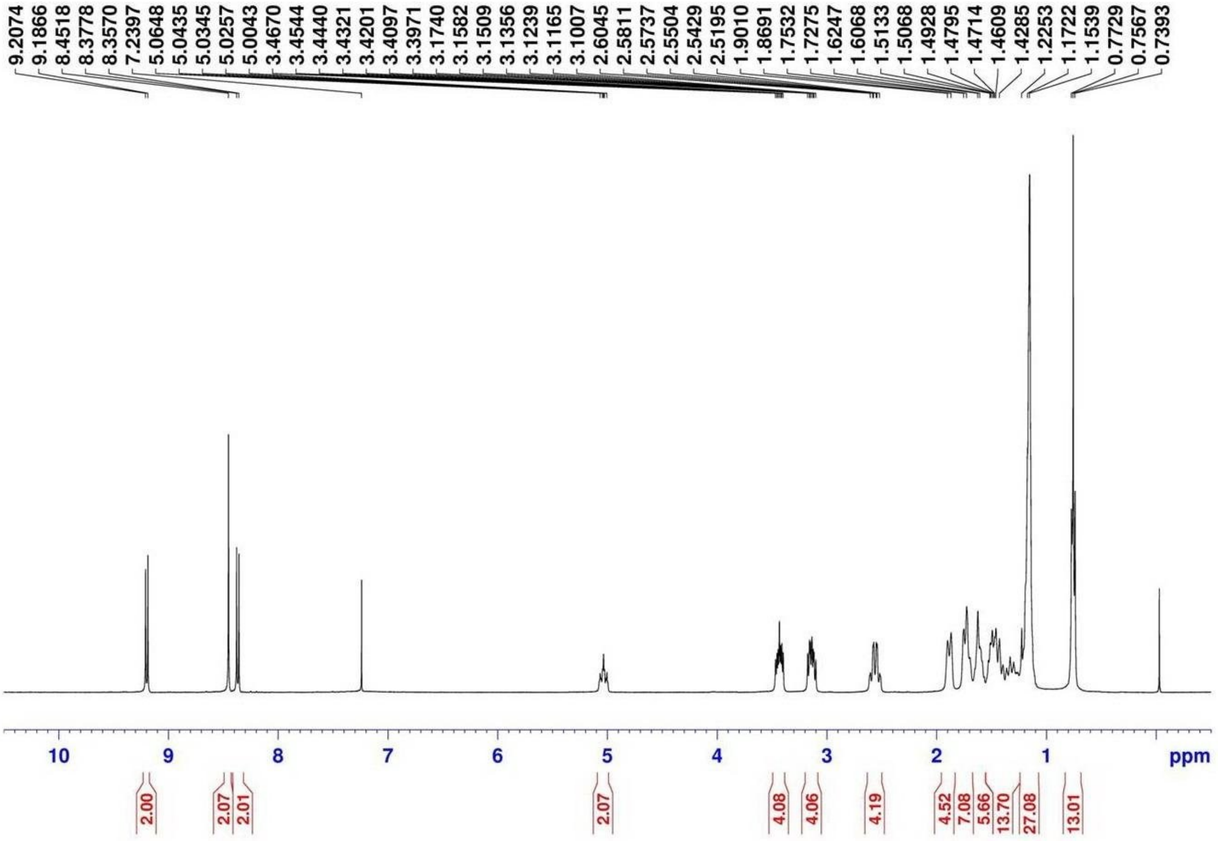


**Figure S6.** $^{1}$H NMR of **1b**.

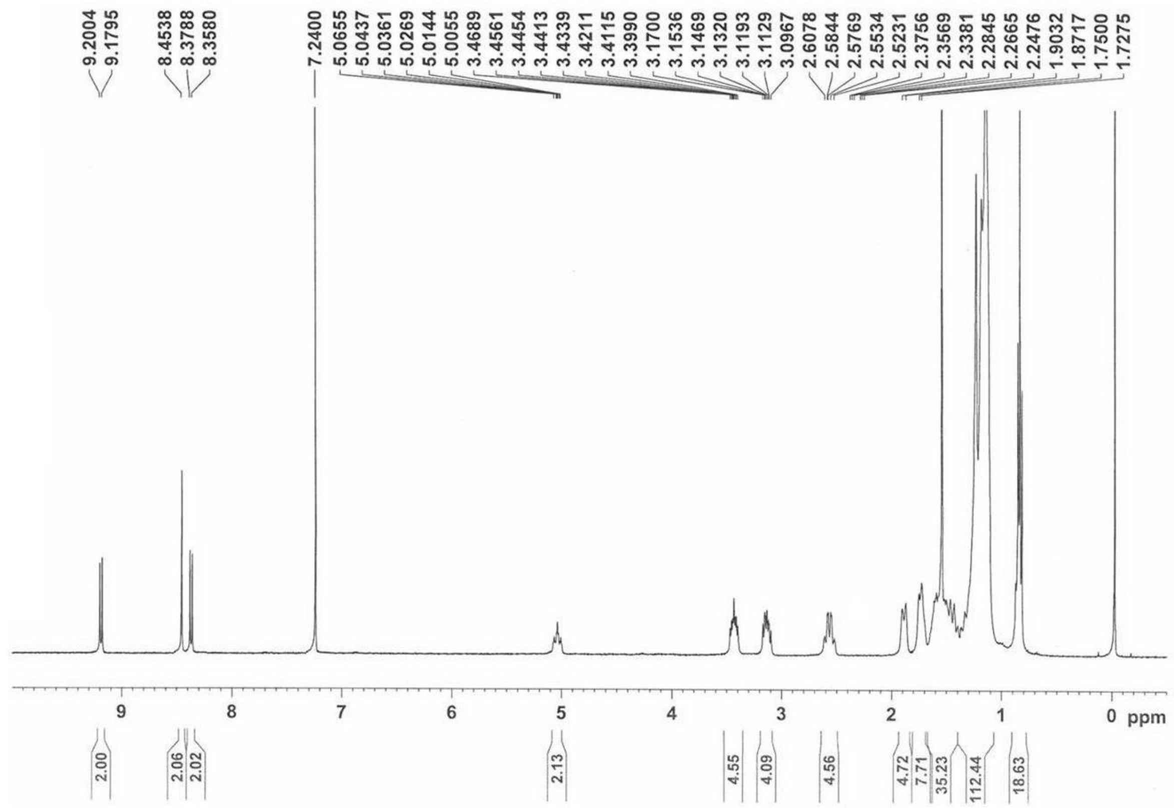

**Figure S7.** $^{1}$H NMR of **1c**.

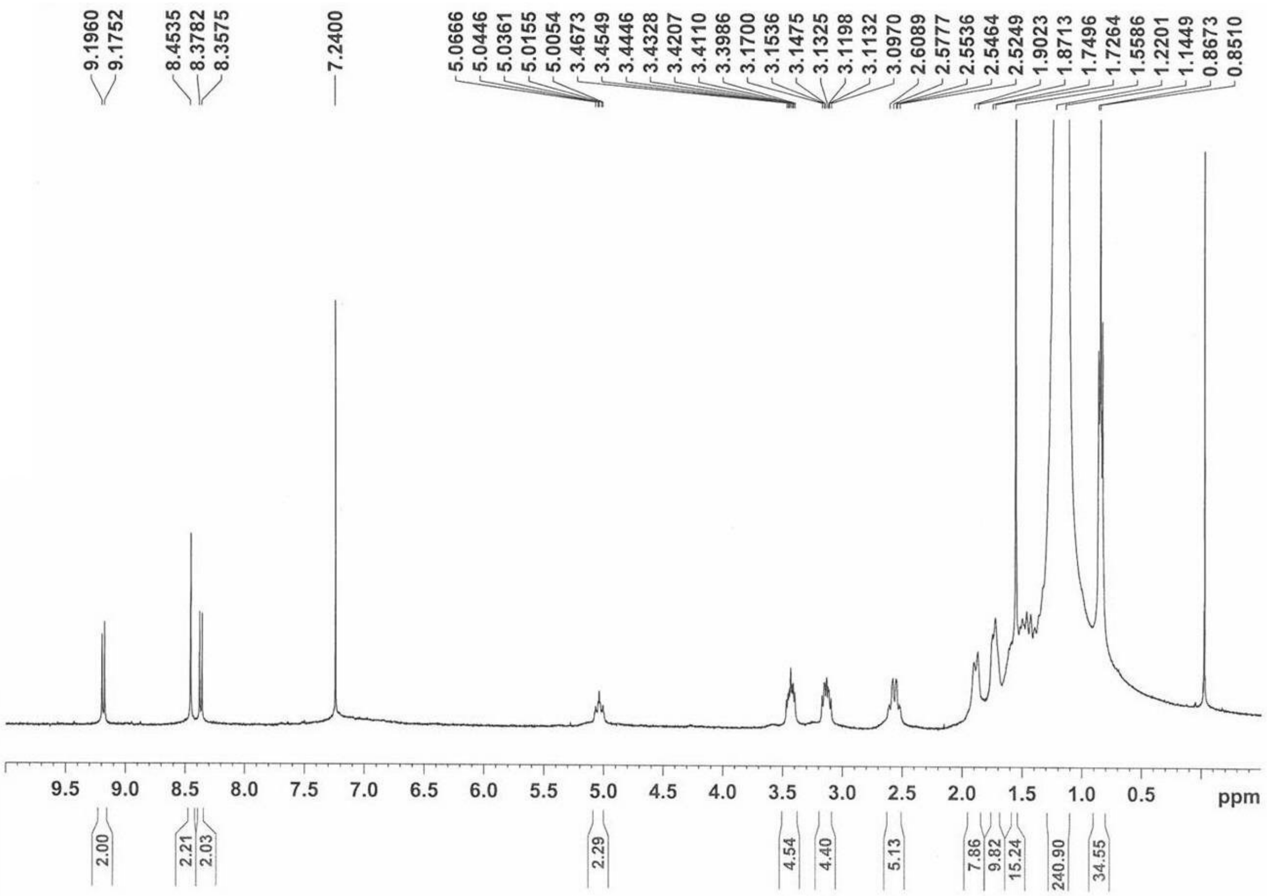


**Figure S8.** $^{13}$C NMR of **1a**.

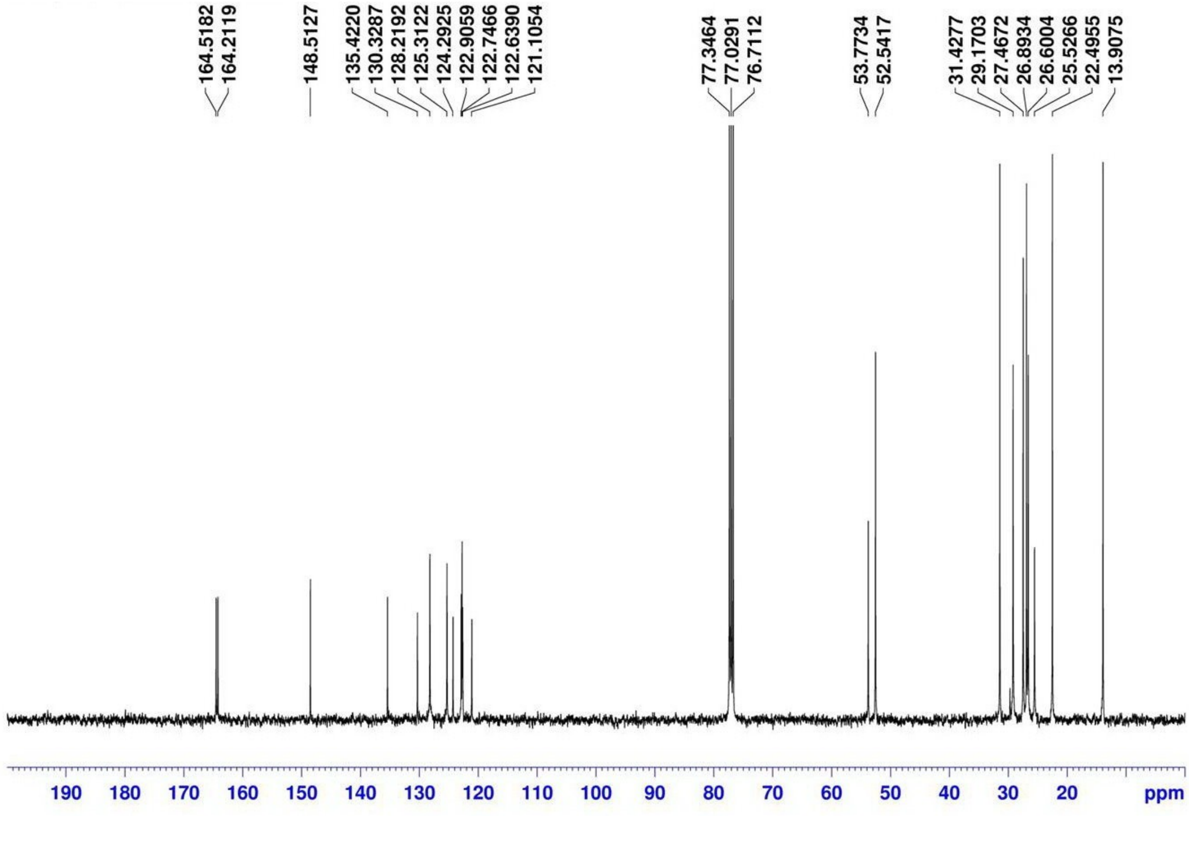

**Figure S9.** $^{13}C$ NMR of **1b**.

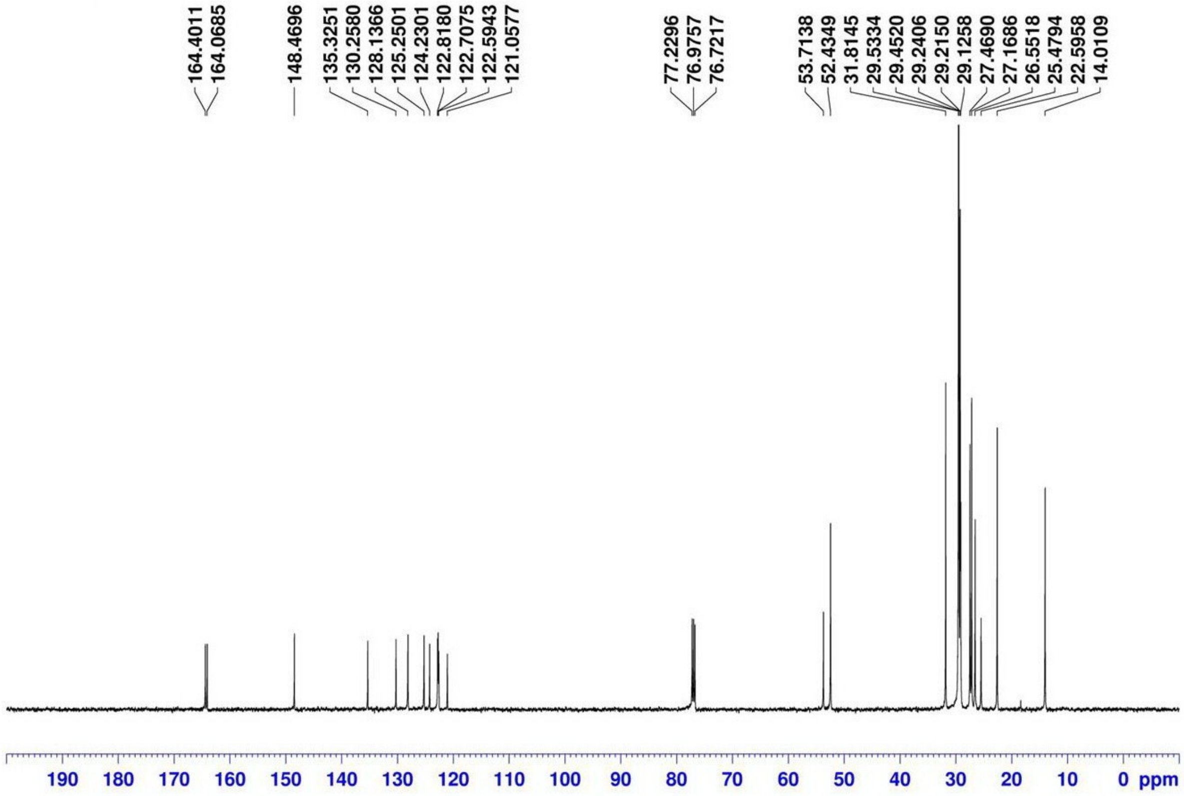


**Figure S10.** $^{13}C$ NMR of **1c**.

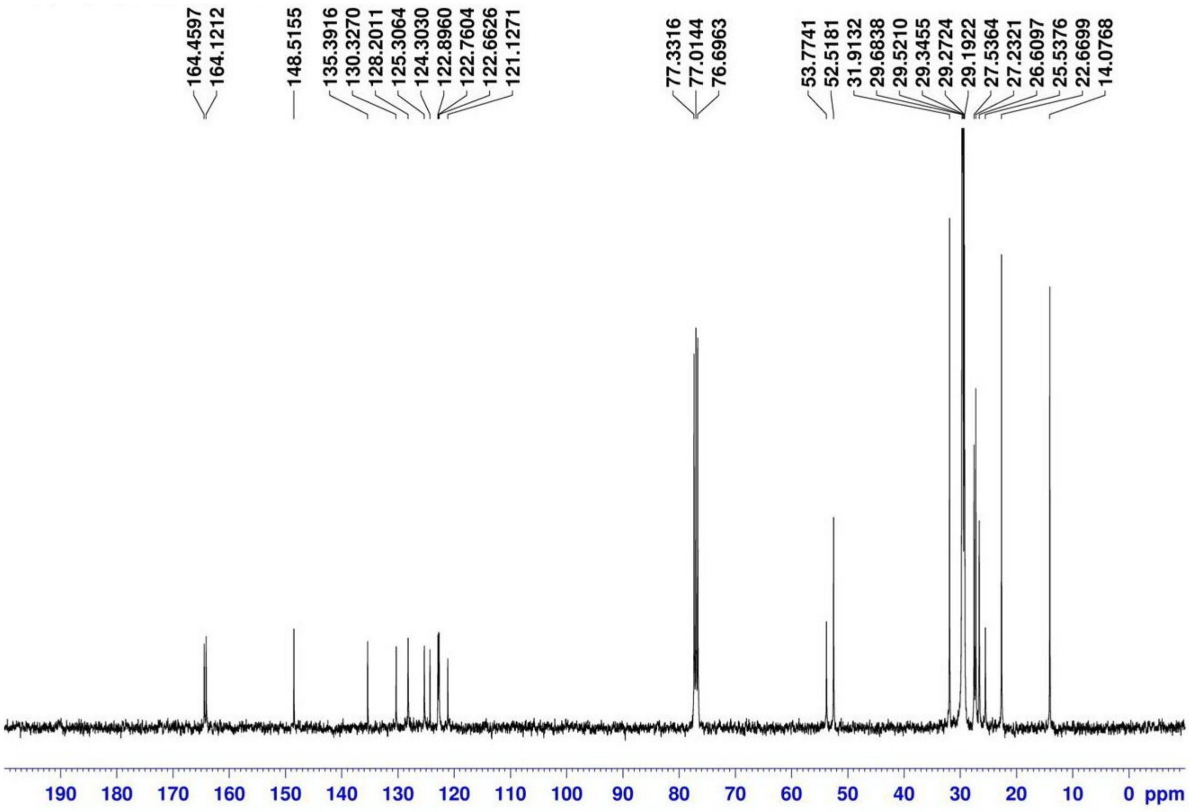

Supplement: Supplementary file 1 [file materials-07-07548-s001.pdf]
